# Outcomes of Pediatric Tracheostomy at Sultan Qaboos University Hospital, Oman: A Multidisciplinary Approach

**DOI:** 10.7759/cureus.115888

**Published:** 2026-09-07

**Authors:** Meetham Al Lawati, Wijdan Al-Hadhrami, Mohammed Al Washahi

**Affiliations:** 1 College of Medicine and Health Sciences, Sultan Qaboos University, Muscat, OMN; 2 Department of Otolaryngology-Head and Neck Surgery, Sultan Qaboos University Hospital, Muscat, OMN

**Keywords:** multi-disciplinary teams, pediatric tracheostomy, tracheostomy complications, tracheostomy outcomes, tracheostomy procedure

## Abstract

Objectives and rationale

Tracheostomy is a surgical procedure associated with a high morbidity rate among neonates and children. Moreover, the decision to perform it is stressful to both parents and physicians, as it is associated with early and late complications and death. However, there are insufficient studies in Oman concerning the outcomes of tracheostomy among children. Therefore, this study aimed to assess the indications, efficacy, outcomes, and complications of tracheostomy performed in children presenting in Sultan Qaboos University Hospital (SQUH), Muscat, Oman, with different indications.

Patients and methods

A retrospective cohort study was conducted among children (< 14 years old) who had undergone tracheostomy in the department of surgery at SQUH between January 1, 2011, and January 1, 2021. Data were collected from electronic patient records from the hospital information system, and descriptive analysis was used to show the results.

Results

A total of 71 children were included in the study. The number of teams involved ranged from two to nine per patient. There were 53 (74.6%) patients who required two to five teams, where 83 (63.8%) complications occurred, while 18 (25.4%) required six to nine teams, where 47 (36.2%) complications developed. The most common early complication was accidental decannulation in 12 (17%) patients, and the most common late complication was colonization by a pathogen occurring in 21 (29.6%) patients. The mortality rate was 26.8% (n=19). The most common cause of death was heart attack/cardiac arrest, where 14 (19.7%) died, followed by 10 (14.1%) deaths due to respiratory failure. The mean duration of hospital stay was 50.18 days (standard deviation (SD) 73.191).

Conclusion

This study shows a reduction in both early and late complications when more teams are involved in the patient’s care. Some of our findings are in line with those mentioned in the literature. This study provided the basis for future retrospective and prospective cohort studies assessing the indications and outcomes of pediatric tracheostomy. We recommend that telemedicine be implemented in the multidisciplinary approach due to its benefits.

## Introduction

Background

Tracheostomy is defined as a surgically created airway made to bypass an obstruction in the upper airway. The management of children with a tracheostomy requires a multidisciplinary team approach. This team includes a speech therapist, pediatricians, otorhinolaryngologists, and the home caregiver [1]. In the past, pediatric tracheostomy was primarily indicated for acute conditions, including trauma and respiratory tract infections. However, with advances in vaccination, particularly against croup, diphtheria, and epiglottitis, as well as improvements in trauma management, neonatal care, and life-support technologies, the indications have shifted toward prolonged endotracheal intubation and upper airway obstruction, including craniofacial malformations, subglottic stenosis, tracheomalacia, and airway tumors [2]. Improved survival among children requiring prolonged mechanical ventilation, particularly those with cardiopulmonary or neurological disorders, has further contributed to this shift. Tracheostomy facilitates pulmonary clearance and may mitigate laryngotracheal injury associated with prolonged intubation. Consequently, prolonged intubation and upper airway obstruction are now among the most common indications for pediatric tracheostomy, accompanied by substantial advances in postoperative care [3].

Pediatric tracheostomy is technically more challenging than adult tracheostomy due to the smaller tube size, softer trachea, and limited surgical space [1]. The risk of tracheostomy-related complications is influenced by patient characteristics and the underlying clinical condition. Early complications may result from tissue trauma, laryngotracheal mucosal injury, or difficult cannula insertion and include bleeding, pneumothorax, pneumomediastinum, and wound complications. Late complications include tracheal or laryngeal stenosis and difficulties with cannula reinsertion. The risk of these complications may be further influenced by factors such as younger age, prematurity, low birth weight, prolonged tracheostomy duration, and underlying comorbidities [4].

Nowadays, many advanced operative techniques reduce morbidity and mortality rates. The most used technique is the regular tracheotomy technique, where a vertical incision in the anterior part of the tracheal wall is performed, and a stay suture is put on one of the sides of the opening. Alternative techniques include horizontal excision by creating an inferiorly based tracheal flap or excising a part of the anterior tracheal wall [5]. It is essential that after tracheostomy, all patients are admitted to the pediatric intensive care unit [6].

The complications linked to pediatric tracheostomy can be divided into early and late complications. One of the most common early complications, which happens mostly within the first week, is accidental decannulation during the repositioning of the child for cleaning or examinations. In addition, there are some late complications like mucus or clot plug deposition within the tube, granulation tissue around the stoma or tube, tracheocutaneous fistula, and stomal/subglottic stenosis due to fibrosis and weakness of the trachea [7, 8].

Pediatric tracheostomy is a potentially life-saving procedure but is associated with substantial morbidity and mortality, with complication rates reported to be 2-3 times higher than in adults [9]. Minimizing these risks requires meticulous anatomical orientation and surgical technique to limit tissue injury, ideally performed by experienced surgeons. Early recognition and prompt management of complications are essential to prevent severe outcomes. The most serious complications include tracheostomy tube obstruction, malposition, and accidental decannulation, which may be particularly life-threatening in critically ill children [6].

Aim of the study and rationale

The tracheostomy procedure has a high morbidity rate among newborns and children. Therefore, there must be proof of upper airway obstruction in the patient before moving forward with the procedure. However, this decision is incredibly stressful to parents and treating physicians due to the long-term consequences and outcomes.

The study aimed to explore the outcome of tracheostomy in children, including the early and late postoperative complications, and assess if those complications are similar or different from those described in the literature.

The multidisciplinary team approach for such cases has been shown to give the correct decision on time and reduce complications. The multidisciplinary team includes neonatologists, pediatric intensivists, pediatric anesthetists, pulmonologists, neurologists, otorhinolaryngologists, social workers, and speech and swallowing therapists.

The specific objectives of this research were to identify the indications and outcomes, including early and late complications of tracheostomy in the pediatric population (< 14 years), at Sultan Qaboos University Hospital (SQUH), Muscat, Oman, assess the efficacy of a multidisciplinary approach in preventing complications, compare the complications among different age groups, assess the survival rate and causes of death in those children after tracheostomy, and suggest ways to implement a multidisciplinary approach by telemedicine easily.

## Materials and methods

Study design, time, and setting

This retrospective study was conducted at SQUH, a tertiary care hospital in Muscat, Oman. The study included pediatric patients from 1^st^ January 2011 to 1^st^ January 2021. 

Data collection

Data were collected using the electronic patient records from the Hospital Information System at SQUH, using the TrakCare® system (InterSystems Corporation, Cambridge, MA, USA).

Patient demographics

The patient demographics collected were sex, age when tracheostomized, tracheostomy insertion (elective/emergency), and the type of tracheostomy. Medical diagnoses like multiple diseases, neurological disease, cardiopulmonary disease, head and neck malignancy, congenital anomalies, metabolic disease, trauma, and others were also recorded.

Outcome and complications

The outcome studied included early complications like accidental decannulation, bleeding, tube obstruction, and surgical emphysema. The late complications studied include colonization by a pathogen, development of granulation tissue, repeated infections, erythema, failure to thrive, swallowing problems, speech problems, and difficult decannulation. The overall mortality rate and the mortality rate among different age groups were also assessed. 

Inclusion and exclusion criteria

The study included all pediatric patients of both genders less than 14 years of age who had undergone tracheostomy in the department of surgery at SQUH. However, we excluded all patients with incomplete or insufficient data about the outcomes.

Ethical consideration

Ethical approval was obtained from the Medical Research Ethics Committee at the College of Medicine and Health Sciences (approval number: MREC #2619) prior to the study.

Data analysis

Data analysis was accomplished using IBM SPSS Statistics software, version 27 (IBM Corporation, Armonk, NY, USA). Categorized variables such as sex, age group, tracheostomy insertion and type, medical diagnosis, number of teams, and causes of death were displayed as frequencies and percentages. Early and late complications were displayed as bar charts. Continuous variables like age and length of stay were presented as mean ± standard deviation (SD), and a histogram for the length of stay. A cross-tab was used to compare the complications between groups involving a different number of teams.

## Results

Patient demographics

A total of 71 children were identified over the 10-year study period. There were 43 (60.6%) male patients. The mean age of patients was 3.32 years (SD ± 3.714), ranging from 0 to 13 years. Forty-one (57.7%) patients underwent tracheostomy insertion before two years of age (Group 1), while 30 (42.3%) were above two years of age when tracheostomized (Group 2). Forty-seven patients (66.2%) underwent emergency tracheostomy insertions compared to the remaining 24 (33.8%) who had elective tracheostomies. The medical diagnosis for each patient was also recorded; there were 28 (39.4%) patients with multiple diseases, 14 (19.7%) with neurological diseases, 10 (14.1%) with cardiopulmonary diseases, four (5.6%) with head and neck malignancies, three (4.2%) with congenital anomalies, two (2.8%) with metabolic diseases, and one (1.4%) with trauma. There were nine (12.7%) patients with other diseases. The patient demographics can be found in Table 1.

**Table 1:** Patient demographics * Includes more than one disease (e.g., neurological disease with congenital anomalies).

Demographics		Number of patients	Percentage of the population (%)	
Sex				
Male		43	60.6	
Female		28	39.4	
Age at tracheostomy insertion				
Below 2 years (Group 1)		41	57.7	
Above 2 years (Group 2)		30	42.3	
Tracheostomy insertion				
Elective		24	33.8	
Emergency		47	66.2	
Type of tracheostomy				
Temporary		53	74.6	
Permanent		13	18.3	
Replacement of tracheostomy tube		3	4.2	
Revision of tracheostomy		2	2.8	
Medical diagnosis				
Multiple diseases*		28	39.4	
Neurological disease		14	19.7	
Cardiopulmonary disease		10	14.1	
Miscellaneous		9	12.7	
Head and neck malignancy		4	5.6	
Congenital‎ anomalies		3	4.2	
Metabolic		2	2.8	
Trauma		1	1.4	

Indications and tracheostomy type

Our study noted that 40 (56.3%) patients required tracheostomy due to upper airway obstruction, followed by 24 (33.8%) with prolonged intubation. The tracheostomy types performed were temporary tracheostomy in 53 (74.6%), permanent tracheostomy in 13 (18.3%), replacement of tracheostomy tube in three (4.2%), and revision of tracheostomy in two (2.8%) patients.

Multidisciplinary team approach

The number of teams involved in the care of pediatric patients was recorded in this study. The multidisciplinary team comprises the ENT team, neurologist/neurosurgeon, pulmonologist, cardiologist, emergency medicine, genetics, paediatrics, maxillofacial surgeon, speech and swallowing therapist, and family counselling. The number of teams involved ranged from two to nine per patient. Group A (n=53; 74.6%) required two to five teams, while Group B (n=18; 25.4%) had six to nine teams (Table 2). 

**Table 2:** Number of teams involved in patient care

Number of teams	Frequency	Percentage (%)
2-5 teams (Group A)	53	74.6
6-9 teams (Group B)	18	25.4
Total	71	100

Figure 1 shows the involvement of multidisciplinary teams in the patients’ care. The findings were as follows: The ENT team was involved in all 71 patients (100%), followed by the emergency medicine team in 59 (81.0%), the paediatrics team in 43 (61.0%), neurology in 41 (58.0%), genetics in 24 (34.0%), cardiology in 22 (31.0%), speech and swallowing therapy in 19 (27.0%), pulmonology in 17 (24.0%), maxillofacial surgery in 11 (15.0%), and family counselling in three (4.0%) patients.

**Figure 1:**
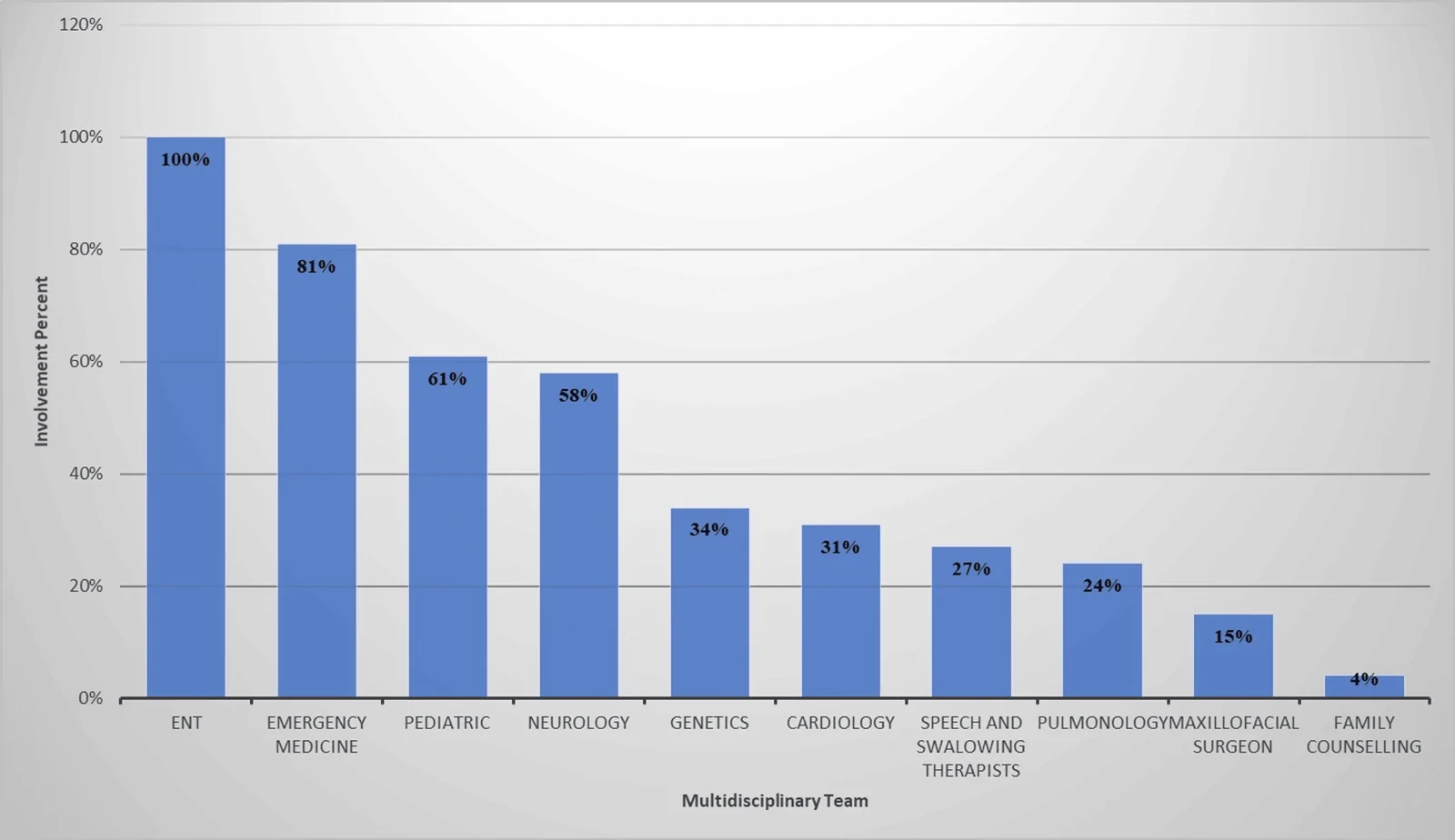
Involvement of multidisciplinary teams in patients care

We divided the number of teams involved in the patients’ care into two groups. Group A involved two to five teams per patient, and Group B involved six to nine teams. We compared the frequency of all complications among the two groups of teams. Accidental decannulation occurred equally in six (50%) patients from each group. Bleeding occurred in seven (100%) patients from Group A compared to 0 (0.0%) in Group B. Tube obstruction in Group A occurred in six (85.7%) compared to one (1.4%) in Group B. Surgical emphysema occurred in five (71.4%) in Group A and two (28.6%) in Group B. Granulation tissue developed in five (71.4%) and two (28.6%) in Groups A and B, respectively. Four patients (66.7%) from Group A had speech problems compared to two (33.3%) from Group B. Swallowing problems occurred in five (55.6%) from Group A compared to four (44.4%) from Group B. Repeated infections occurred in nine (60.0%) from Group A compared to six (40.0%) from Group B. Failure to thrive occurred in seven (70.0%) from Group A compared to three (30.3%) from Group B. Colonization by pathogen occurred in 13 (55.6%) from Group A compared to eight (44.4%) from Group B. Difficult decannulation occurred in two (40.0%) from Group A compared to three (60.0%) from Group B. The total frequency of complications in Group A was 83 (63.8%) compared to 47 (36.2%) in Group B. These findings are shown in Table 3. 

**Table 3:** Number of teams and complications

	Number (percentage (%) with complication)	
Complication	Group A (2-5 teams) (n= 53)	Group B (6-9 teams) (n= 18)
Accidental decannulation	6 (11.32%)	6 (33.33%)
Bleeding	7 (13.21%)	0 (0.0%)
Tube obstruction	6 (11.32%)	1 (5.55%)
Surgical emphysema	5 (9.43%)	2 (11.11%)
Granulation tissue	14 (26.41%)	6 (33.33%)
Speech problems	4 (7.55%)	2 (11.11%)
Swallowing problems	5 (9.43%)	4 (22.22%)
Repeated infections	9 (16.7%)	6 (33.33%)
Failure to thrive	7 (13.21%)	3 (16.66%)
Colonization by pathogen	13 (24.53%)	8 (44.44%)
Difficult decannulation	2 (3.77%)	3 (16.66%)
Erythema	5 (9.43%)	6 (33.33%)

Overall early and late postoperative complications

We identified four early postoperative complications in 33 patients (46.5%) and eight late complications in 50 (70.4%). The most common early complication was accidental decannulation in 12 (16.9%) patients, followed by bleeding in seven (9.9%), tube obstruction in seven (9.9%), and surgical emphysema in seven (9.9%). The most common late complication was colonization by pathogen, occurring in 21 (29.6%) patients, followed by the development of granulation tissue in 20 (28.2%), repeated infections in 15 (21.1%), erythema in 11 (15.5%), failure to thrive in 10 (14.1%), swallowing problems in nine (12.7%), speech problems in six (8.5%), and difficult decannulation in five (7.0%) patients (Figures 2, 3). 

**Figure 2:**
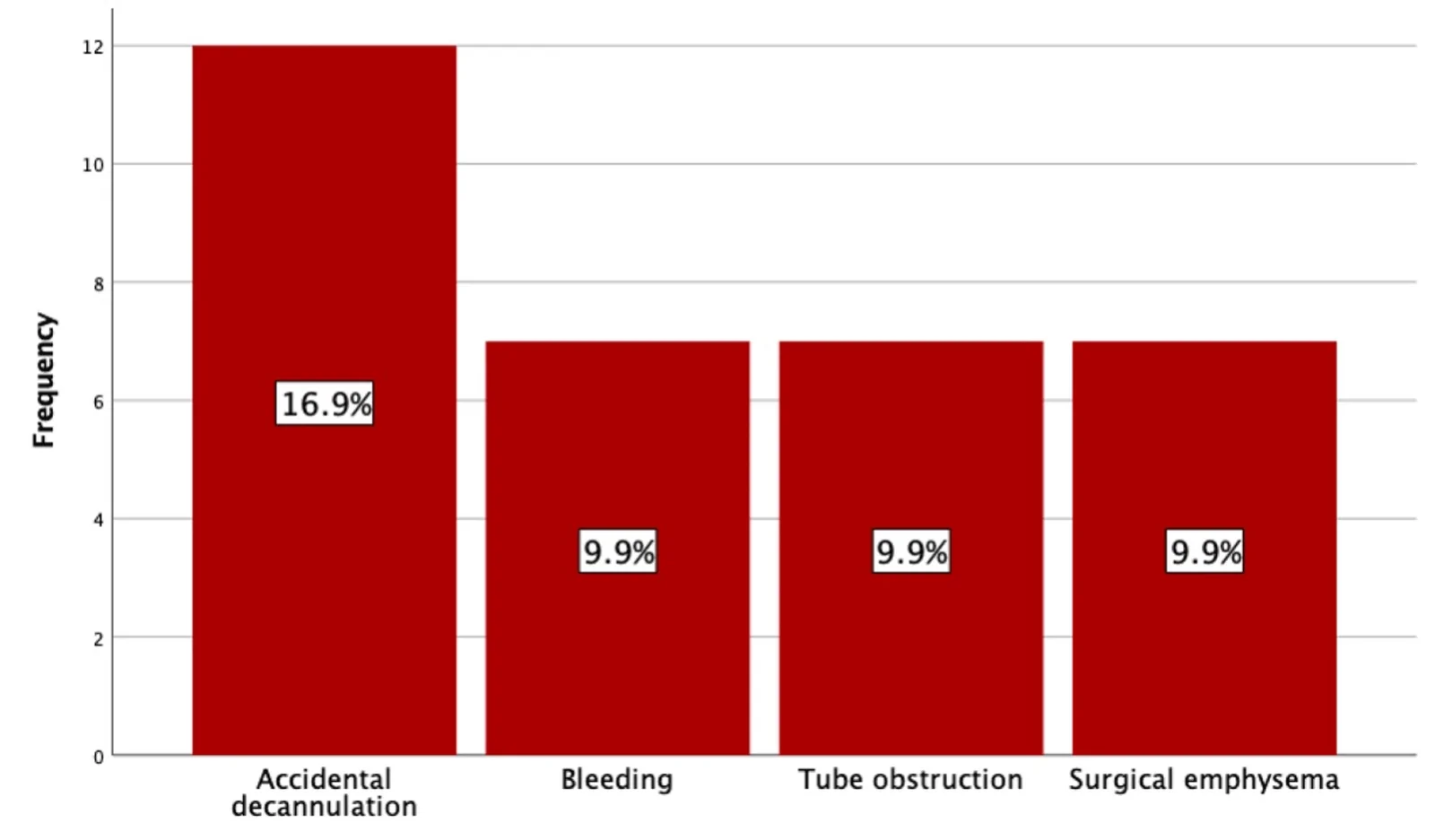
Overall early complications following tracheostomy

**Figure 3:**
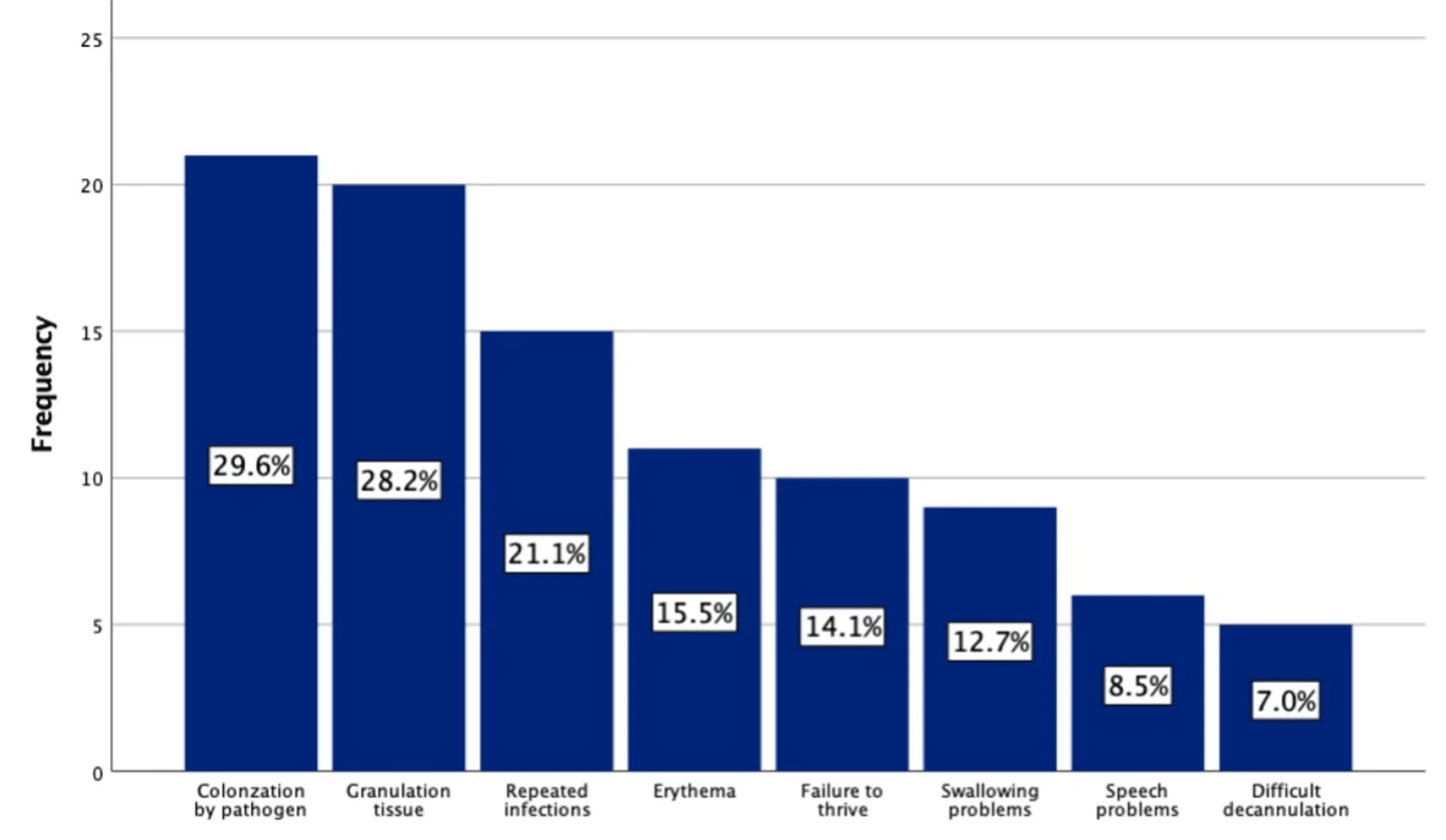
Overall late complications following tracheostomy

Early and late postoperative complications among age groups

We compared the early and late complications between the two age groups. Group 1 included children less than two years old, and Group 2 had children above two years old when they were tracheostomized. Group 1 had accidental decannulation as the most common complication in eight (11.3%) patients, while bleeding was the most common early complication in Group 2, occurring in six (8.5%) patients. The second most common early complication in Group 1 was surgical emphysema in three (4.2%) patients compared to Group 2, which was tube obstruction in five (7.0%). The third most common complication in Group 1 was tube obstruction in two (2.8%) patients compared to Group 2, which had surgical emphysema and accidental decannulation in four (5.6%) each. Bleeding was the least common complication in Group 1, which occurred in one (1.4%) patient (Figure 4). 

**Figure 4:**
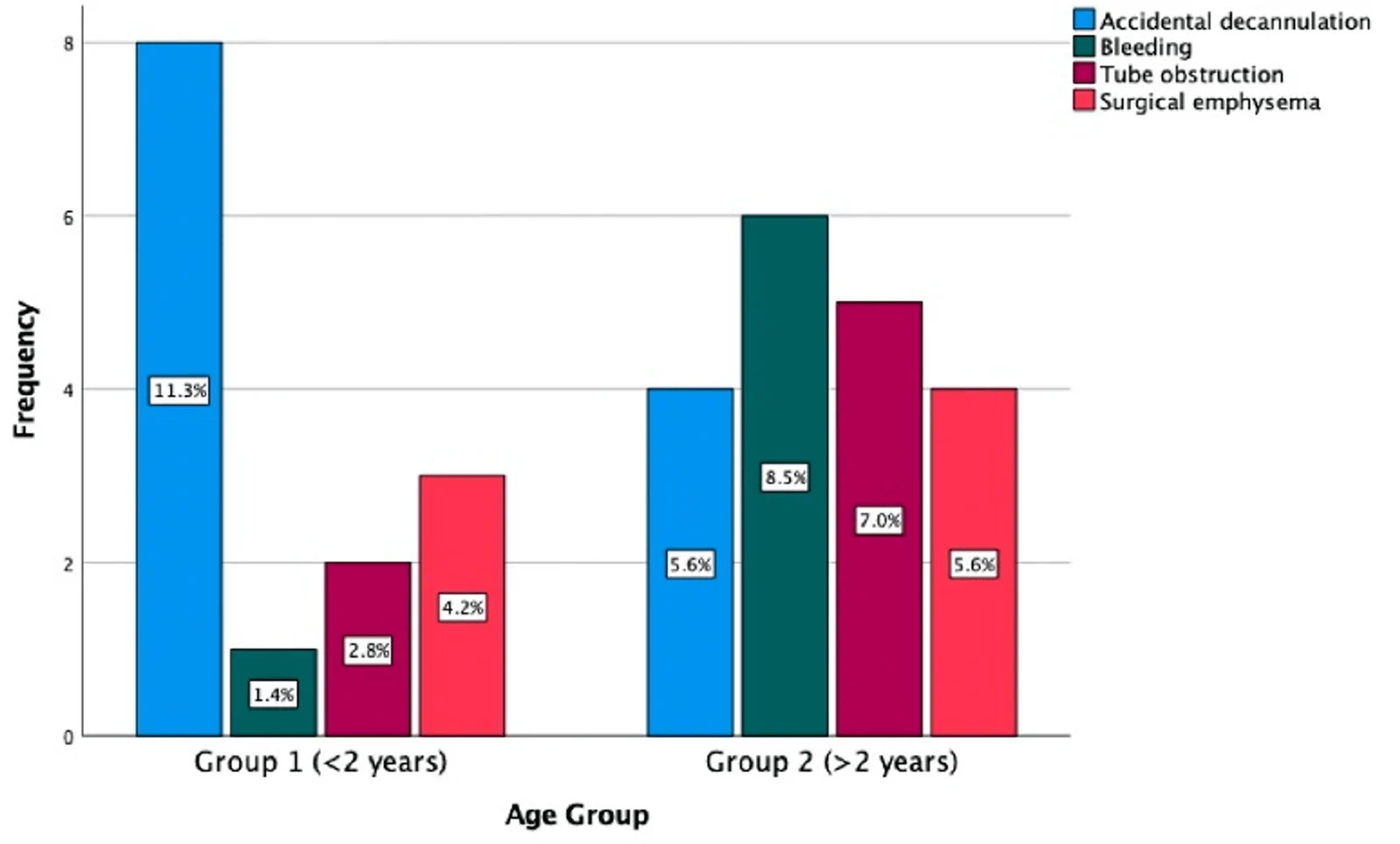
Early complications following tracheostomy in age groups

We studied eight late complications and compared them between the two groups. The most common late complication in Group 1 was colonization by a pathogen in 17 (23.9%) patients compared to granulation tissue in 13 (18.3%). The second most common late complication in Groups 1 and 2 was repeated infections in nine (12.7%) and six (8.5%). The third most common late complication in Group 1 was erythema and failure to thrive in equal frequencies in eight (11.3%) compared to Group 2, which had swallowing problems and colonization by a pathogen in equal frequencies in four (5.6%). Granulation tissue in Group 1 was the fourth most common late complication in seven (9.9%), compared to Group 2, which had erythema and speech problems in equal frequencies in three (4.2%). The fifth most common late complication in Group 1 was difficult decannulation and swallowing problems in equal frequencies in five (7.0%), compared to failure to thrive in two (2.8%) in Group 2. The least common late complication in Group 1 was speech problems in three (4.2%) patients. There were no cases of difficult decannulation in Group 2 (Figure 5).

**Figure 5:**
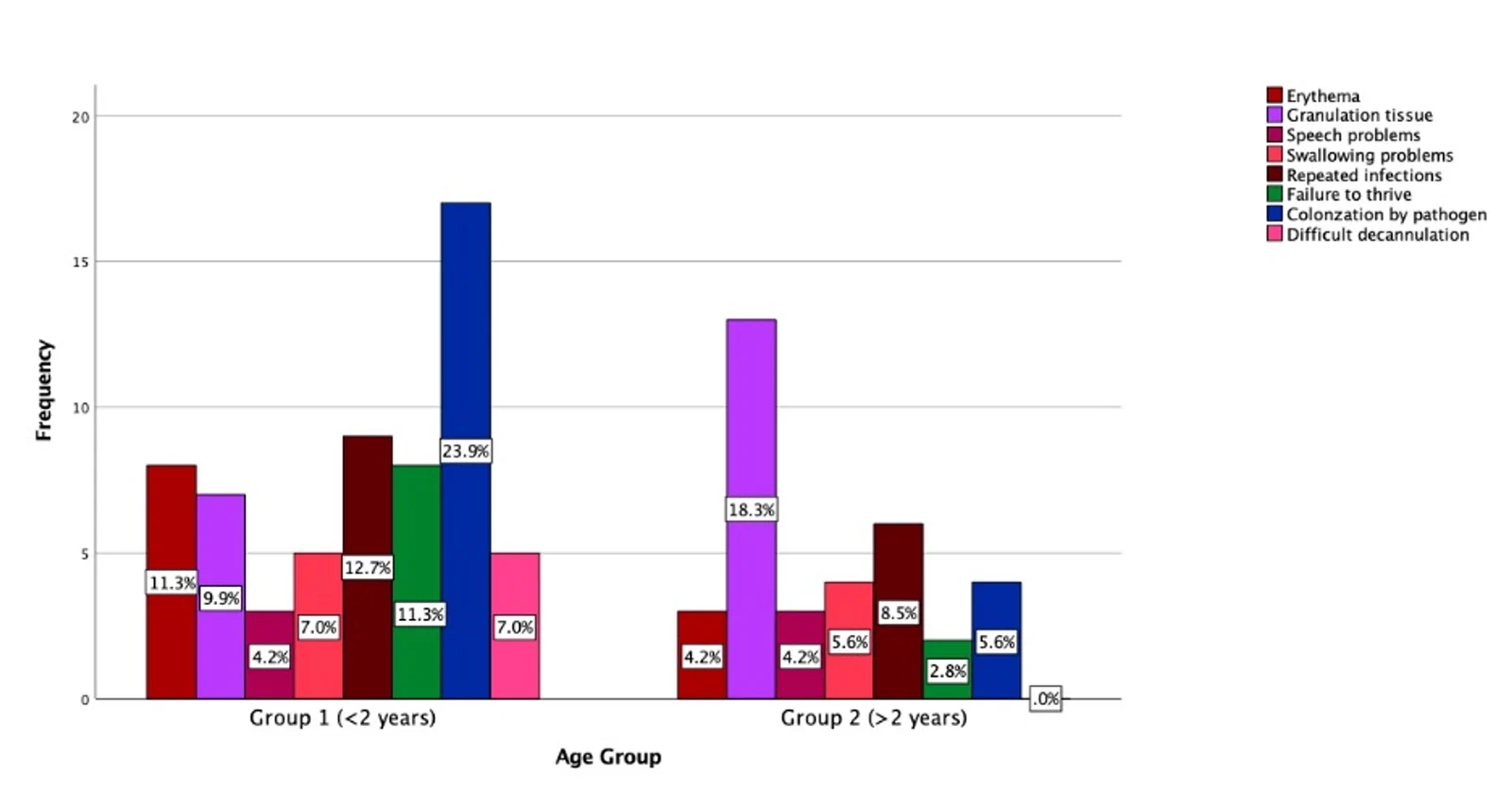
Late complications following tracheostomy in age groups

Length of hospital stay

Our study population's mean duration of hospital stay was 50.18 days (SD 73.191). The length of stay in the hospital ranged from one to 392 days. A histogram of our population's duration of stay is shown in Figure 6.

**Figure 6:**
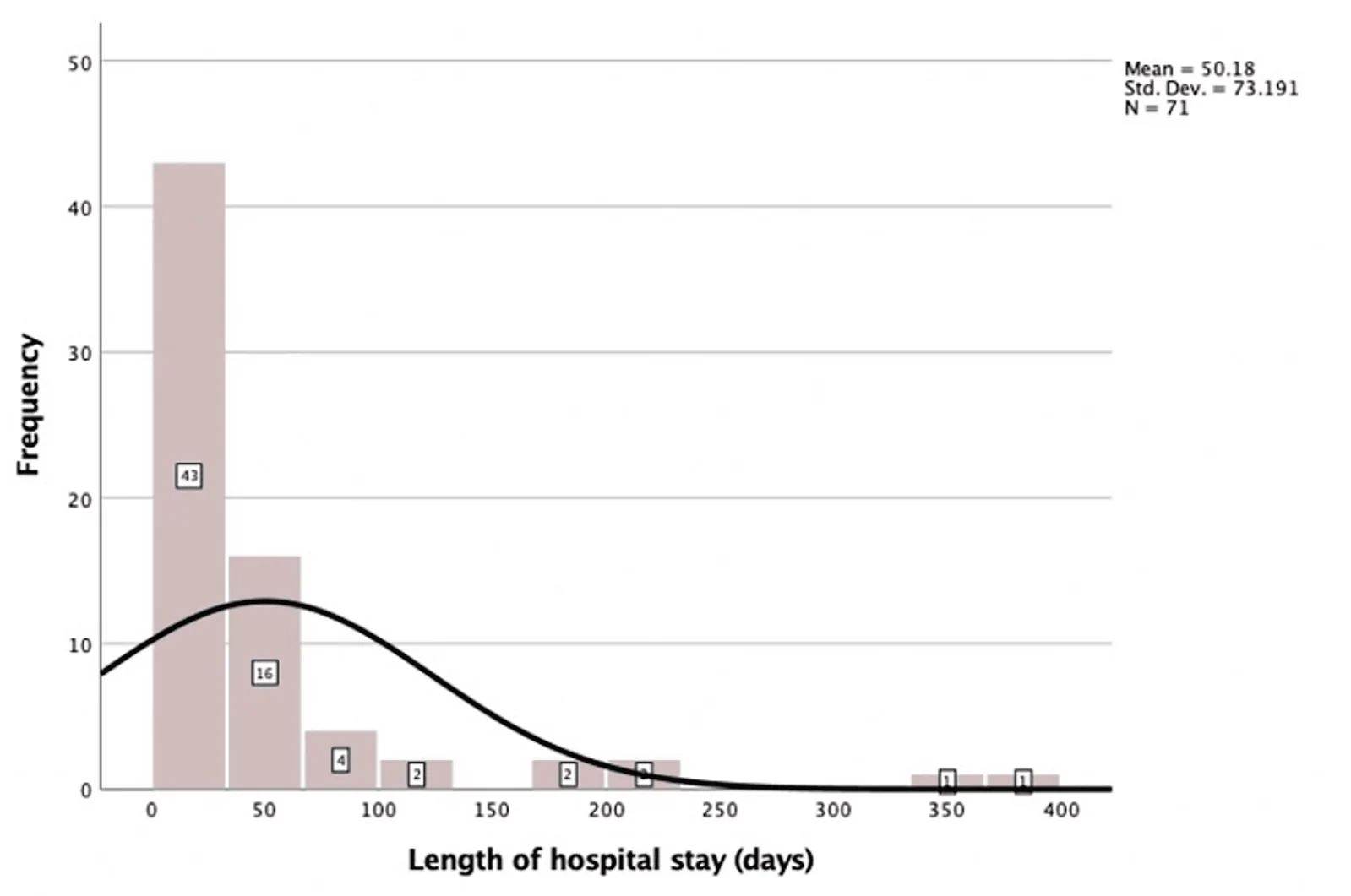
Histogram of length of stay Std. Dev.: standard deviation

Overall cause of mortality

The overall mortality rate of the children who underwent tracheostomy at our center was 26.8% (n=19). Six deceased patients (8.5%) were from Group 1, compared to 13 (18.3%) from Group 2. The most common cause of death was heart attack/cardiac arrest, where 14 (19.7%) died, followed by 10 (14.1%) deaths due to prolonged hypoxia/respiratory failure and one (1.4%) death, each due to accidental decannulation and tube obstruction.

Causes of mortality among age groups

Heart attack/cardiac arrest in Groups 1 and 2 was the most common cause of death in eight (11.3%) and six (8.5%) patients, respectively. The second most common cause of death in both groups was respiratory failure, with six (8.5%) and four (5.6%) patients in Groups 1 and 2, respectively. In Group 1, accidental decannulation and tube obstruction caused death in one (1.4%) patient, respectively (Table 4).

**Table 4:** Causes of death among age groups

Cause of death	Number of patients	Percentage of deceased patients, Percentage of cohort (%)
Respiratory failure		
Below 2 years	6	31.6, 8.5
Above 2 years	4	21.1, 5.6
Heart attack/cardiac arrest		
Below 2 years	8	42.1, 11.3
Above 2 years	6	31.6, 8.5
Accidental decannulation		
Below 2 years	1	5.3, 1.4
Above 2 years	0	0,0
Tube obstruction		
Below 2 years	1	5.3, 1.4
Below 2 years	0	0,0

## Discussion

This study evaluated the indications and outcomes of pediatric tracheostomy at SQUH. The most common primary indication was upper airway obstruction in 40 patients (56.3%), followed by 24 (33.8%) patients with prolonged intubation. Previous studies show prolonged ventilation as the most common indication (range 20%-61%), followed by subglottic stenosis (5.2%) [7]. The difference can be explained by the fact that airway obstruction includes many causes, including subglottic stenosis [10].

The outcome included early and late postoperative complications, survival rate, and causes of death among different age groups. Compared to the literature, we report an early complication rate of 46.5% in 33 patients, which reports 7.4% to 11.2% [7]. Our significantly higher rate can be explained by more than one early complication in one patient and the majority being diagnosed with multiple diseases. In keeping with the literature, our study's most common early postoperative complication was accidental decannulation in 12 (16.9%) patients, followed by bleeding, surgical emphysema, and tube obstruction in seven (9.9%) cases in the total population. The literature shows that the most common early complication is accidental decannulation, with an occurrence rate of 3.9% (six of 153), followed by bleeding at 3.3% (five of 153) [7]. Our higher rates could be due to the placement of incorrect tube sizes, errors during the fixation of the tube, poor nursing care, and insufficient physicians' education. 

The late complication rate in our population was 70.4%, occurring in 50 patients. The most common late complication was colonization by a pathogen, occurring in 21 (29.6%) patients, followed by the development of granulation tissue in 20 (28.2%) patients. The literature reports granulation tissue as the most common late complication in 19.6% of patients with late complications [7]. The small difference is due to our relatively smaller sample size.

Roberts et al. report that only 6.4% were emergency tracheostomies [7], while our study shows that the majority of the cases in 47 patients (66.2%) were performed as an emergency compared to 24 (33.8%) patients who were tracheostomized electively. This may be due to the high incidence of upper airway obstruction in our population as an indication for the procedure, where it is considered an emergency, and the patient must be treated immediately.

The number of children in our study who died after the procedure was 19 of 71 children (26.8%). This mortality rate is similar to that reported in the literature (15%-39.2%). The most common causes of death in our population were heart attack/cardiac arrest and respiratory failure. Ten patients (14.1%) died of respiratory failure in our population, compared to seven of 172 (4.1%) who died of the same cause [7]. In addition, one patient (1.4%) died of accidental decannulation, and another patient (1.4%) died of tube obstruction. Deaths in our population due to heart attack/cardiac arrest are due to coexisting morbidities and acute events, and they are not related to the tracheostomy tube per se. Deaths due to tube obstruction could be related to the smaller tube size, the tube touching the wall of the trachea, and the presence of a clot. To prevent obstruction and eventual death, the tube's position should be readjusted or replaced with a longer one, and the nurses should be educated on the proper handling of tube obstruction by a clot. 

Our study focused on the role of the multidisciplinary team in patients' care. As seen in Table 3, both the early and late complications occurred in higher frequencies in patients treated by two to five teams than in patients treated by six to nine teams. Bleeding, tube obstruction, surgical emphysema, granulation tissue, speech problems, swallowing problems, repeated infections, failure to thrive, and colonization by pathogens occurred more frequently in Group A (two to five teams) than in Group B (six to nine teams). On the other hand, accidental decannulation, difficult decannulation, and erythema occurred more frequently in Group B (six to nine teams). An important note is that the slightly higher rate in Group B is due to the difference in only one patient (n=1) each. Our results showed fewer complications when more teams are involved, which aligns with previous literature findings of decreased complications [11].

Unfortunately, only parents of three (4.2%) patients received family counselling. This could be due to a lack of documentation or a possible follow-up in other Ministry of Health hospitals. Another reason could be the difficulty of coming to the hospital, where many Omani families live far away from our center. At SQUH, parents with a tracheostomized child are provided with leaflets, line communications, and online instant messaging services to the treating physician. However, there is a big space for improvement, which can be done using a telemedicine system.

Over the last decade, telemedicine, or communication technology, has risen [12]. However, the practice guidelines for ENT specialists limited in-person care during the COVID-19 pandemic. Due to the pandemic, there was a need to expand telemedicine by converting clinic visits to virtual consultations. The use of telemedicine in the multidisciplinary approach in tracheostomized patients is discussed below.

Tracheostomy tubes are an open site for droplet exposure, and patients who need to be tracheostomized usually have other diseases that increase their risk of infection from in-person care. As a result, individuals with tracheostomies are excellent candidates for telemedicine appointments.

When a specialized tracheostomy nursing team is available, multidisciplinary visits for patients generally include pediatric ENTs, pulmonologists, and speech-language therapists. Providers should assess the patient's airway to reach a diagnosis before the decision to perform tracheostomy is carried out. Healthcare professionals may be able to determine a respiratory rate and should urge caregivers to get an oxygen saturation value using a home monitor, if available, for objective assessments of respiratory status. Caregivers effortlessly arranged their child supine on a level surface and configured the video equipment according to provider instructions in their experience. Healthcare professionals could view the stoma with caregiver manipulation of the tracheostomy tube. Then, they could assess and adjust the ventilator settings under virtual guidance.

To test airway patency and voice, providers instructed caregivers during the virtual consultation to occlude the tracheostomy tube using a finger when the patient attempts to vocalize on expiration. Then, under the virtual supervision of a speech-language pathologist, a speaking valve or capping trial can be done. Providers and healthcare professionals should get counselling and training on implementing a wearing schedule and any contraindications to usage if they are considered acceptable candidates [13].

Given the past information, we must start implementing telemedicine in our center so that patients can easily follow up with the multidisciplinary team and get appropriate guidance regarding their children's care. In addition, the use of telemedicine will save time and money. However, limitations to be considered are the low internet speeds in rural areas in Oman and the initial cost of setting up a new system in the hospital.

Limitations

This study was conducted in a single center, resulting in a small sample size and, therefore, not reflecting the outcome of all tracheostomies with fair distribution. Also, the nature of the retrospective study affects the data collected, with some missing results and a loss to follow-up for the outcome. There was almost no follow-up for the patients regarding decannulation, so it was not mentioned in our study. In addition, our study only recorded the most common complications: four early and eight late, while a similar study had 16 early and 72 late complications.

Recommendations and impact of research

To our knowledge, this is the first research that studies the outcomes of pediatric tracheostomy in Oman. So, this study provided the basis for other research projects in this field.

It is recommended that telemedicine be implemented in the multidisciplinary approach because of its many advantages, including time and money savings, with possibly better patient care.

Future retrospective studies, including more centers in Oman, are needed to have a fairer distribution of patients among the different regions and a more extensive study population. Also, a prospective study is recommended for better results since we will see the importance of multidisciplinary teams.

Clinically, the study helps healthcare providers by showing the importance of the involvement of several teams to come up with the best decision for the patient and reduce early and late complications.

## Conclusions

In conclusion, some of our findings align with those mentioned in the literature. Our study shows a reduction in early and late complication rates when more teams are involved in patient care. Although the study results cannot be generalized, it has provided a base for further retrospective and prospective cohort studies describing the indications and outcomes of tracheostomy in Oman. Future studies, including multiple centers, are required to compare institutional practice, identify more areas for practice improvement, and build on the current evidence base. We recommend that telemedicine be implemented in the multidisciplinary approach due to its benefits.
